# An Essay on the Pathology and Treatment of Cancer

**Published:** 1873-10-15

**Authors:** N. Senn

**Affiliations:** Ashford, Wisconsin


### Original Communications.

### AN ESSAY ON THE PATHOLOGY AND TREATMENT OF CANCER.

BY N. SENN, M.D., ASHFORD, WISCONSIN. READ BEFORE THE ROCK RIVER MEDICAL SOCIETY, AND PUBLISHED BY REQUEST.

(*Continued from last Number.*)

The varieties of cancer may be arranged in the following groups, according to the proportions of their anatomical constituents:

1. Epithelial.
2. Scirrhus.
3. Encephaloid.
4. Colloid.
5. Melanotic.

1. The epithelial variety is not included by all authors under the head of cancer, on account of its supposed semi-malignancy. Siebert applied to it the name of cancroid. Its lesser fatality and liability to reproduction after extirpation consists in its more superficial location, and, as a rule, its earlier submittance to a thorough operation.

If left to itself, it eventually destroys life as certainly as any other variety. It consists almost exclusively of cells; hence it never forms tumors of any size. It ulcerates early, and destroys the tissues around it with remarkable rapidity.

We find it on the surface of the skin or mucous membranes.

2. Scirrhus, as its name implies, consists of a tumor of cartilaginous density, owing to the predominance of the fibrous stroma over the cellular elements.

Its most favorable location is the female breast. It seldom affects a young person, being more particularly a disease of the period of involution.

3. Encephaloid cancer is very vascular, and of rapid growth. It is composed of a delicate stroma and numerous large cells.

It attacks, most frequently, the eye and antrum. As a rule, young persons are more subject to it than to scirrhus.

4. Colloid, or alveoler, cancer is composed of a very delicate network of fibrous tissue, enclosing enormous round or cellular cells, and a peculiar gelatinous, amorphous material so characteristic of this variety of cancer.

It is of very rare occurrence, and seldom forms distinct tumors, but more frequently is diffused over the surface which it affects.

5. The principal feature of melanotic cancer is the deposition of pigment molecules in its substance, giving it a brown or black color. It occurs in many localities simultaneously, is very malignant, and, happily, seldom met with.

*Artiology.*—The essential cause of cancer is still unknown. Many suggestions have been made, and numerous theories advanced, but they have all failed to establish their correctness by a sufficient number of facts. The humeral pathologists sought for the cause in an impure morbid condition of the blood, regarding the local disease merely as an effect. This doctrine meets the approval of many, even to-day. Scientific researches into the minute pathological and histological processes tend more and more to prove that its origin is local, the general condition following as a secondary result. The following facts tend strongly to substantiate the local theory: 1. Cancer affects persons of apparently robust health, who may remain so frequently for years, showing constitutional symptoms only after the local disease has made considerable advance. 2. As a rule, only one primary tumor is present. 3. The secondary deposits usually occur in proximity of the primary tumor, in the lymphatic glands connected with it.

If the local disease were only symptomatic, the constitutional disturbance ought, necessarily, always to precede it; and more likely, a number of deposits would take place simultaneously in different parts of the body.

C. J. M. Langenbeck* believes that the syphilitic dyscrasia stands in a direct causative relation to cancer. Notwithstanding this eminent authority, I should hesitate to accept his views, as in the majority of cases that I have seen this element could be safely excluded.

The believers in the constitutional origin

* Langenbeck's Chirurgie Band, v. Seite, 793.

mention its hereditary tendency as one of their principal arguments in its favor. In quite a number of cases, the hereditary tendency is obvious; but the same can be said of congenital deformities, mental peculiarities, and non-malignant tumors. Age exercises a positive influence in determining the frequency with which it occurs; while no age, even the fœtus in utero, is exempt. It manifests a preference for the period of involution —after the thirty-fifth or fortieth year.

Occupation may act as a predisposing cause. It is a fact well-established that chimney-sweeps are prone to suffer from epithelial cancer of the scrotum, the accumulation of soot in its folds being a constant source of irritation. Long-continued irritants of any kind, as mechanical, chemical, or pathological, under certain circumstances, may so alter the vitality of the parts on which they act as to establish a carcinomatous process.

Waldeyer claims that certain simple tumors may, under favorable circumstances, assume a malignant character, leading rapidly to local destruction and general infection.

Climate and the geological conformation of the surface appear to exercise some influence in the causation of cancer. They deserve to be studied with more care than heretofore, to determine their exact influence.

Depressing mental emotions are mentioned by most authors as indirect causes; but their influence in causing other affections is equally well established. In former times, direct inoculation was believed to be possible. The experiments of Dupuytren, Birtt, and Alibert, yielded only negative results.

B. V. Langenbeck claims to have produced cancer in the lungs of animals, into whose veins he had injected fresh cancer juice.

The frequency of cancer appears to increase with the advance of civilization.

*Diagnosis.*—In the different diagnosis of cancer, we have to consider non-malignant and other malignant neoplastic tumors. At a time when cancer ought to be recognized for its successful treatment, it is extremely difficult to do so. In general practice, a positive diagnosis is usually made only after the tumor has progressed to extensive infiltrations, and so, by its clinical course, has plainly demonstrated its dangerous character. The history of the case, the physical properties of the tumor, and the microscopical appearance of its cellular elements, should all be examined carefully, to enable us to make an early and correct diagnosis. To consider the differential diagnosis between all benign tumors and the different varieties of cancer, in detail, would be superfluous. We will only briefly refer to sarcoma, which, in malignancy, forms the counterpart of cancer. We shall succeed best if we compare them in tabular form:

| CANCER. | SARCOMA. |
|---|---|
| 1. Tumor never attains a very large volume; it is irregular in shape, and firmly attached to surrounding parts. | Tumor sometimes very large; regular in shape; movable; and showing no disposition to usurp the space of other organs. |
| 2. Tumor undergoes early destructive charges by degeneration or gangrene. | Tumor retains its vitality for a long time, on account of its more favorable vascular supply. |
| 3. Structure composed of a stroma, epithelial cells, and an inter-cellular substance. | Structure more homogeneous, being composed mainly of connective tissue cells. |
| 4. Metastatic deposits occur generally in the course of lymphatic vessels. | Metastatic deposits, almost without exception, follow the course of the blood-vessels. |

They resemble each other in producing general symptoms, and in their liability to return after extirpation.

*Prognosis.*—The prognosis of cancer is, in the majority of cases, a grave one, from the fact that, generally, when we are called upon to treat it, it has so extensively infiltrated the neighboring tissues as to render its perfect removal difficult, if not impossible. The least dangerous form is the epithelial variety. A fatal termination is hastened if the disease is located in some of the vital organs, if its growth is rapid, and if it affects young individuals. It may prove fatal in six months, or may extend over a period of ten, or even twenty, years, as in some rare cases of epithelial cancer or scirrhus of the breast.

The average duration may be estimated at three years. With many patients it would be advisable to keep them in ignorance as long as possible as to the true character of their disease, in order to prevent those mental depressions which follow such a knowledge, and necessarily hasten the final termination.

*Treatment.*—It would be very natural to expect that, in the treatment of such an obstinate disease, almost every known remedy should have had a trial. The honest physician has administered his remedies with a consciousness of uncertainty as to their ultimate effects, and has almost invariably been disappointed; while the charlatan, with positive assurances of success, has found ample opportunity to deceive the pitiable victims of this terrible disease. The remarks of Artius, following his graphic description of uterine cancer, prove, even to-day, only too true: "*Proinde affectio harc incurabilis est, velut etiam Hippocrates promnciavit: mitigari tamen ac lenivi potest.*"

Various remedies, as mercury, chloride of gold, arsenic, iodine, iron, cicute, belladonna, calendula, cundurango, etc., have been given at different times, with the vain hope to eradicate the supposed cancerous diathesis; but they have all failed in turn to exercise any specific curative effect whatever. Mercury was one of the main ingredients of the famous Decoctrino Zittmanni, once so much in use among the older German physicians for all constitutional diseases.

Rush had an unflinching confidence in the efficacy of arsenic as a remedy for cancer, given internally and applied externally. He claimed that it was as much a specific for cancer as mercury for syphilis.

Dr. Washington Atlee, of Philadelphia, at the last meeting of the American Medical Association, in St. Louis, stated that arsenic, if given for a long time, positively prevents the return of cancer, after a thorough operation for its removal.

Prof. N. S. Davis, of Chicago, has seen good results from the long continued use of arsenic in cancers of the breast. He generally combines conium with the arsenic. With the history of cundurango you are all familiar; that it is one of the great humbugs of the past, we are all satisfied. The fact is, we are not in possession of a single known remedy, if administered internally, that will exert the least effect in arresting the progress of this disease; and if our views of its pathology will be found to be correct, it is doubtful whether we *shall* ever be fortunate enough to find one.

If it is in its incipiency a local disease, we must naturally resort to local means for its successful extermination. For it is here we meet the enemy face to face—that cruel one, before whom so many thousands have died a tenfold death.

The local treatment may be divided as follows:

1. Palliative.
2. Refrigeration.
3. Starvation.
4. Pressure.
5. Injections.
6. Caustics.
7. Excision.

1. The palliative treatment is to be resorted to if the disease is located in an organ that will not admit of removal without immediate danger to life; also, if the disease has advanced beyond the reach of the knife; or if it is complicated with another disease fatal in its tendency.

It consists in keeping the part affected at rest; the removal of any accidental inflammation, or any source of irritation; subduing pain, and the use of disinfectants, to correct the fœtor of the discharges.

As local anæsthetics, conium, stramonium belladonna, opium, and carbolic acid, have been used.

If the skin remains intact, a plaster may be used with great advantage, into which conium, stramonium, or belladonna, enter largely. If ulceration has already occurred, a combination of morphine with carbolic acid, glycerine and water will be found the most beneficial. As disinfectants merely, a weak solution of carbolic acid, permanganate of potassa, or chloride of lime, may be used.

An ointment, made by boiling the leaves of stramonium in lard, is recommended very highly by Prof. N. S. Davis, of Chicago.

2. The application of intense cold was first introduced into practice by Dr. James Arnott. He used a mixture of common salt and ice, applied for five or six minutes at a time, and repeated frequently during the day.

He claimed for it both palliative and curative properties. While it temporarily removed pain, the author himself confesses that the disease generally progresses to its final fatal termination.

3. To arrest the vigorous development of the tumor, both general and local means have been resorted to to diminish its nutritive supply.

The general treatment consisted in withholding nutritious food, limiting the patients to the plainest articles of diet.

This starvation cure was practiced extensively by the older German physicians; but on the whole, I believe it yielded very unfavorable results, and has consequently been almost entirely abandoned.

Local starvation consists in cutting off the blood supply to the parts by ligating its nutrical arteries.

Dr. Markoe * reports several cases of malignant disease of the superior maxillary bones, where decided improvement followed ligation of the carotid artery.

Where the removal of the whole tumor is impossible, this operation in exceptional cases is advisable and practical. As a curative means, it of course cannot be relied upon.

4. Compression by means of adhesive plaster and bandages was first used by Young, in the Middlesex Hospital, more than forty years ago. The practice was afterwards revived by Racemier, in France, and Arnott, in England. Arnott used an elastic air-cushion, in order to equalize the pressure more perfectly. In different hands it has met with varying results, but on the whole it has failed to fulfill the expectations of its most earnest advocates, even in the hands of the most experienced. If pressure would have the effect to cause a retrograde degeneration, rendering the neoplastic deposit capable of absorption, it would be the great desideratum in the treatment of cancer; but it is doubtful whether such a favorable result was ever obtained; on the contrary, I believe, in the majority of cases,

* Markoe on Diseases of the Bones, p. 395 et sequi.

by adding another source of irritation, it accelerates the growth of the tumor.

5. The fact that acetic acid destroyed the nuclei of the cancer-cells, induced Mr. Brodbrent to apply it in practice. With a hypodermic syringe he injected dilute acetic acid into the interior of a tumor, repeating the operation at intervals, until the whole mass had sloughed away, leaving a clean, granulating surface. It is left for the reader to come to his own conclusions whether it ultimately healed.

Dr. Charles H. Moore,* in his remarks on the use of acetic acid, condemns its use in primary cancer, but favors its use in secondary tumors.

Dr. Dieu has derived much benefit from the local use of strong acetic acid to epithelial cancers of the face.

The great objections to the hypodermic use of any remedy will always be: 1st. To limit its action to the cancer cells without affecting the healthy structures; 2d. The impossibility by this means to reach every cell in the tumor.

6. Those that have regarded cancer as a local disease, have, from times immemorial, sought for remedies to effect its destruction. Almost every substance capable of destroying the tissues has been used. The chief articles, however, have been the following: arsenic, chloride of zinc, caustic potassa, chloride of bromine, and the red, hot iron.

Prof. Rust recommends, in strong terms, the application of arsenic, according to the formulæ of Frere Cosme:

℞.—Arsenici albi, ℈ii.

Cinnabaris artefacte, ʒ ii.

Sanguinis draconis, grs. xii.

Cineris solearum calceamentorum, grs. viii.

M. fl. pulv.

He claims that the *combination* constitutes its power; by leaving out any of its ingredients he has found, by actual experience, its efficacy impaired. †

Dupuytren found caustic potassa, equally

* Bright, on Cancer, p. 82.

† Rust's Clinical Lectures, p. 415.

efficacious with arsenic in destroying superficial cancers.

Dr. Bright, in his recent little work "On Cancer," recommends chloride of zinc as the best caustic.

He has four preparations: No. 1 consists of solid extract podophyllum, one part; pure chloride zinc, three parts; starch, one-fourth part; red saunders, one-fourth part; water, sufficient quantity to form a thick paste. No. 2 consists of a saturated aqueous solution of chloride of zinc. No. 3 is a paste, like No. 1, with the exception of using carbolic acid in the place of water. No. 4 is simply an arrow made of pure chloride of zinc, with starch, and dried at a temperature of 212° F. Armed with these remedies he claims to have *cured* 90 per cent. of cancer. Such an assertion must be regarded with suspicion as long as the most eminent authorities speak of it as "*a disease that never forgives*,"

I do not doubt the authors sincerity, but the correctness of his diagnosis.

In looking over carefully his reported cases, it becomes apparent that many of them, according to his own description, were not cancer, but lupas; in another part of his work he says that lupus and cancer are one and the same disease. This explains his many wonderful cures.

I will not attempt to give a review of his work; suffice it to say, that his pathological views of the disease are fifty years behind the time; and the description of his treatment, with the report of cases, sounds, to say the least, egotistical. Pulling out a cancer by its roots, without destroying any of the surrounding healthy tissues, after applying any caustic, is not compatible with the scientific status of the nineteenth century.

Dr. Landolfi, an Italian physician of great reputation, employed a mixture of equal parts of chloride of bromine, zinc, gold, and antimony, mixed with a sufficient quantity of flour to form a viscid paste. He also administered the chloride of bromine internally, in doses of one-tenth of a grain; and applied a wash of the same of ten to twenty drops to a pint of water. His bold applications, repeated over and over, resulted, in many instances, in a cure; but that it failed in the hands of most of them that tried it after him, is certain, from the fact that, at present, it is but seldom, if ever resorted to.

Of all the caustics, the red-hot iron is the oldest and perhaps the most reliable, when the location of the disease renders it at all admissible. The method of application has of late been much improved by the introduction of the galvanic cautery. With the galvano-caustic wire, the morbid mass is removed, as with a knife, without any hæmorrhage. Its only disadvantage consists in the establishment of an eschar, rendering primary union impossible.

7. The knife is one of the oldest remedies. Men of science have ever been its most devoted friends. Quacks have universally condemned its use, and done most towards creating the existing popular prejudice against its legitimate employment.

This is very natural, as its use requires an accurate anatomical knowledge of the parts to be operated upon, while the application of caustics is a bloodless procedure, and on that account eagerly sought after by ignorant pretenders.

The all-important local indication to be fulfiled consists in removing, if possible, *every particle* of diseased structure. We are at present not in possession of a single caustic that has a specific destructive action on the cancer-cell. All destroy the tissues with which they come in contact, without regard to their structure.

I claim for the knife the following advantages: 1st. With it any desirable amount of tissues can be removed at once. 2d. It leaves the wound in a condition to heal by the first intention, without necessarily exciting suppurative inflammation. 3d. The operation is free from pain, as the patient may be put under the influence of an anæsthetic. 4th. If the tumor involves any large vessels, the hæmorrhage can be immediately arrested, while under such circumstances, the application of caustics is liable to be followed by troublesome or even fatal hæmorrhage after the separation of the slough. The cause of failure, in the majority of cases, consists in not removing all of the neoplastic deposits during the operation. It is a very deceptive growth, infiltrating itself further than is generally supposed, gaining at the same time early entrance into the lymphatic vessels.

*The one and great rule, then, always should be to operate early and thoroughly.* The line of incision should extend beyond the limits of the disease into the apparently healthy tissues, and, if possible, in the direction of the course of the lymphatic vessels. If only part of an organ is affected, it should be removed entire. Great care should be exercised, during and after the operation, not to inoculate the surface of the wound with cancer-cells. It would be advisable to wash its surface with a solution of carbolic acid, to destroy any remaining germs, if any doubt exists as to their existence, before closing the wound. If possible, the wound should be healed by the first intention. Any means to establish suppuration, for the purpose of destroying any remaining portions of the tumor, should not be resorted to, as the irritation necessarly excited by them could not fail to hasten a return of the morbid process.

The following may be regarded as indications for an operation:

1. If all the diseased tissues can be removed without any immediate danger to life, and no secondary symptoms have appeared, or no internal deposits taken place. This includes those cases where lymphatic deposits have taken place, but are accessible to the knife.

2. If the pressure caused by the tumor compromises the function of important organs, as the larynx, trachea or vagus nerve.

3. In some cases, as a palliative to remove temporarily pain, or a sloughing offensive gangrenous mass.

4. When, recurring hæmorrhages endanger the patients life, and ligating the principal arterial trunk is insufficient to arrest it.

Contra-indications are secondary internal deposits; too great debility; extreme old age; and the co-existence of other fatal diseases.

That metastatic deposits in internal organs are more apt to follow an operation, than if the disease is not interfered with, experience has not established. In most of the cases, the recurrence takes place in, or about, the old cicatrix, and is owing to an imperfect removal of the disease at the time of operation.

CONCLUSIONS.

1. Cancer is an atypical epithelial neoplasma.

2. It is primarily a local disease.

3. In its earliest stages it is amenable to local treatment, and becomes incurable after the system becomes infected.

4. An early and thorough excision affords the most reliable means to remove the parts affected and prevent constistutional contamination.

